# Hyperspectral Imaging Reveals High Water and Hemoglobin Content at Rest and Decreased Oxygen Levels After Physical Activity at the Residual Limb of Non‐Dysvascular Lower Limb Amputees

**DOI:** 10.1111/micc.70051

**Published:** 2026-01-18

**Authors:** Luis A. Pardo, Charlotte Brinkmeyer, Meike A. Wilke, Lisa Lorbeer, Marc Varel, Arndt F. Schilling, Jennifer Ernst

**Affiliations:** ^1^ Hannover Medical School Department of Trauma Surgery Hannover Germany; ^2^ Department of Trauma Surgery, Orthopedic Surgery and Plastic Surgery University Medical Center Göttingen Göttingen Germany; ^3^ Faculty of Life Sciences, Hamburg University of Applied Sciences (HAW) Hamburg Germany; ^4^ Department for Sport Psychology Institute for Sport Science, Johannes Gutenberg‐University Mainz Mainz Germany

**Keywords:** edema, hyperspectral imaging, major limb amputation, microcirculation, residual limb health, skin, ulcers, venous stasis

## Abstract

**Objective:**

Skin integrity is one factor determining residual limb health. Oxygen deficiency caused by energy consumption and/or mechanical stress is the most common reason for skin breakdown at the residual limb (RL), limiting physical activity and causing residual limb pain (RLP). This study aims to detect differences in microcirculation at rest (T1) and after walking for 6 min (T2) with a socket prosthesis at different zones at the residual limb (RL) and the corresponding areas at the sound limb (SL).

**Methods:**

Amputation and RLs' demographics as limb circumference (LCF), subcutaneous fat thickness (SCFT) and microcirculatory parameters as tissue oxygenation saturation (StO_2_), near‐infrared perfusion index (NIRPI), tissue hemoglobin index (THI) and tissue water index (TWI) were visualized and analyzed at different zones of the SL and RL of ten non‐dysvascular major lower limb amputees at T1 and T2 using ((HIS), TIVITA Tissue Diaspective Vision, Germany).

**Results:**

LCF and SCFT were lower at the RL than at the SL. A significant reduction of NIRPI after walking was observed at location F2, affecting both limbs. StO_2_ decreased significantly from T1 to T2 only at the residual limb at AL, with no corresponding change in the sound limb.

**Discussion:**

These findings demonstrate localized exercise‐related reductions in oxygenation at the distal RL, whereas microcirculation of the SL remained unchanged. Underlying factors and a possible impact on the overall residual limb health need further investigation.

**Abbreviations**
3D: three‐dimensional
6MWT: 6‐minute‐walk‐test
AAS: amputee activity score
AL: amputation level
HRQoL: health related quality of life
HSI: hyperspectral imaging
ISPO: International Society for Prosthetics and Orthotics
LCF: limb circumference
MCFL: Medicare's Functional Classification Level
NIRPI: near infrared perfusion index
pts.: points
RL: residual limb
RLP: residual limb pain
SCFT: subcutaneous fat thickness
SL: sound limb
StO_2_
: tissue oxygenation saturation
TF: transfemoral
THI: tissue hemoglobin index
TT: transtibial
TWI: tissue water index

## 1 Introduction

Residual limb health is a prerequisite for continued use of the prosthetic device. The residual limb (RL) serves as the interface between the amputee and the prosthesis. Impaired blood supply leads to reduced oxygen supply. The role of microangiopathy in the pathogenesis of ischemia and the interrelationship between macro‐ and microangiopathy of the lower extremities are not well established [1]. The rate of postoperative perfusion‐related complications in amputee care is reported to be as high as 40% [2]. Postoperative perfusion disturbances jeopardize tissue viability and disturb and prolong wound healing with possible following tissue necrosis and infections, which frequently require revision surgeries [3, 4, 5]. Revision surgery after amputation is associated with increased morbidity and mortality, longer hospitalization, and higher healthcare costs [6, 7].

At the healed RL, oxygen deficiency can be caused by energy consumption and/or mechanical occlusion of the skin capillaries. Together, they are the most common reasons for skin breakdown at the residual limb (RL) limiting physical activity. Local microcirculatory parameters at the different zones of the RL are difficult to quantify and visualize before complications develop.

Furthermore, ischemic conditions can decrease mobility and rehabilitation due to premature muscular fatigue and increased risk of developing ischemic‐driven amputation‐associated pain [8, 9, 10]. Overall, reduced tissue perfusion of the residual limb (RL) can significantly impair residual limb health.

A few microcirculatory studies of the residual limb have evaluated different conditions, such as consumption during physical activity and external compression of the capillary bed when wearing a liner.

One limitation of these studies has been that the methods used therein measured and visualized local tissue oxygenation at the RL at an area limited to mm due to the design and dimension of the sensors used. Hyperspectral imaging (HSI) is a new, non‐invasive, non‐ionizing, and contactless imaging modality of microcirculatory tissue oxygenation based on spectrometric characteristics providing diagnostic information about the tissue physiology, morphology, and composition after assessing light spectra of different wavelengths reemitted by molecules with individual remission rates. Thus, the color‐coding during HSI of the RL provides planar information about oxygenation or ischemia at different zones of superficial tissue layers at the RL still at a high resolution [11, 12, 13]. Technical details have been published recently [11, 14, 15, 16, 17]. Several studies have shown that HSI detects skin tissue oxygenation and perfusion deficits that correlate with pathological changes of global markers of tissue oxygenation [18, 19, 20, 21].

This study aims to analyze microcirculation parameters at different zones of the sound limb (SL) and residual limb (RL) of non‐dysvascular lower limb amputees at rest and their microcirculatory reaction (−capacity) after walking in a socket prosthesis.

We hypothesize that walking in the socket prosthesis, the RL will exhibit decreased oxygen levels, while the SL will demonstrate stable microcirculatory parameters. We expect that HSI will reveal notable differences in microcirculatory parameters between the residual limb (RL) and the sound limb (SL) in non‐dysvascular lower limb amputees.

## 2 Materials and Methods

### 2.1 Subjects

Unilateral transfemoral (TF) and transtibial (TT) amputees between 18 and 80 years after giving informed consent were included. Dysvascular amputations as well as persons with neuromuscular diseases or diseases affecting the gait and peripheral neuropathies were excluded. The study was approved by the local ethics committee (App. No. 26/03/18).

### 2.2 Data Collection

#### 2.2.1 HSI Imaging

The hyperspectral camera system TIVITA Tissue (Diaspective Vision, Germany), which operates in the spectral bandwidth from 500 (visible) to 1000 nm (near‐infrared) was used within the study. At 50 cm distance the three‐dimensional (3D) data cube with two spatial and one spectral dimension implemented in the HSI camera generated a planar image of 16 × 11.5 cm within 6 s [11, 12, 22, 23]. The following parameters could be retrieved from the image: tissue oxygenation saturation (StO_2_) [0%–100%], near‐infrared perfusion index (NIRPI) [0–100], tissue hemoglobin index (THI) [0–100], and tissue water index (TWI) [0–100]. The ratio of oxygenated to deoxygenated hemoglobin was used to determine the relative oxygen saturation and its distribution in superficial tissue areas (StO_2_: approximately < 1 mm depth) and in deeper tissues areas (NIRPI: approximately 3–5 mm depth) [23, 24]. The THI describes the existing hemoglobin distribution in the analyzed field and can indicate venous stasis. TWI describes the water distribution in the analyzed field and can indicate edema [11, 12, 13, 18, 19, 25].

### 2.3 Experimental Setup

**FIGURE 1:**
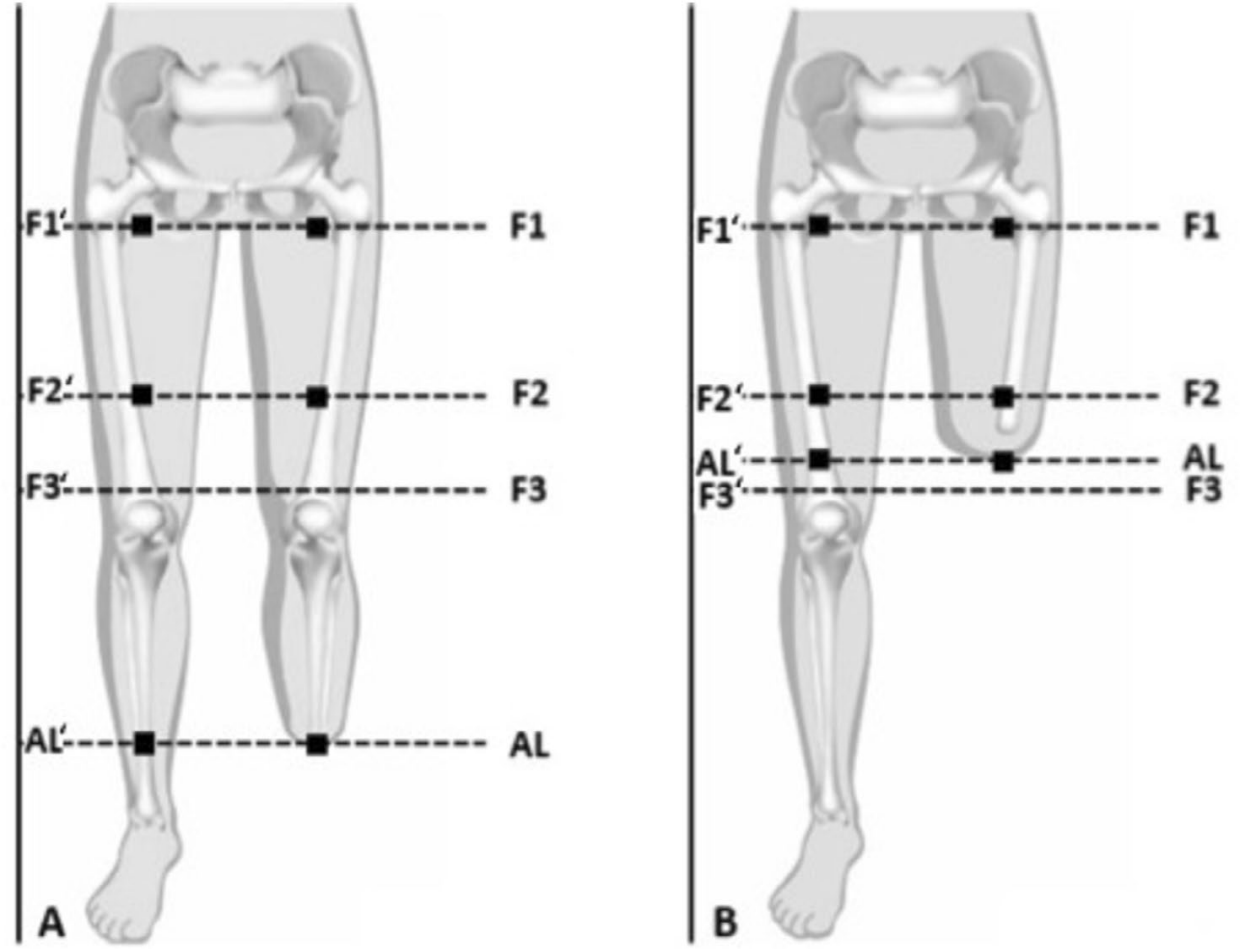
Localization of measurement points F1, F2 and AL in TT amputees (A) and TF amputees (B) at the RL and corresponding measurement points at the SL indicated with '. F1 = 30 mm distal of tuber ossis ischi; F2 = middle between F1 and F3; F3 = 20 mm proximal to the knee joint, auxiliary point; AL = most distal point at the RL. Note: F1, F2, and AL lie inside the socket for TF amputees (B), whereas only AL lies inside the socket of TTs.

The camera was mounted perpendicularly at a fixed distance of 50 cm. To avoid interference with the camera's integrated illumination system, ambient light was minimized by dimming room lights and shielding the measurement area so that no external light reached the limb during image acquisition [26]. Images were taken at defined positions F1, F2, F3, AL (Figure 1) on the SL and on the RL at rest (T1, or “Pre Exercise”) and after physical activity (T2, or “Post Exercise”) consisting of a 6‐minute‐walk‐test (6MWT). T1 was defined after a 10‐min rest phase, in which the patients were encouraged to lie relaxed in supine. T2 was measured immediately after the 6MWT and the prosthetic socket was removed from the residual limb immediately thereafter. The measurement points (F1, F2, F3, AL) at the SL and RL were determined according to an established measurement protocol for socket fabrication (Ottobock SE & Co. KGaA) (Figure 1).

#### 2.3.1 Anthropometric Data

Limb circumference (LCF) was measured at predefined anatomical landmarks (F1, F2, and 0.5 cm proximal to the most distal point of the residual limb, AL). Corresponding contralateral points (F1′, F2′, AL') were used on the sound limb. Subcutaneous fat thickness (SCFT) was measured with a skin fold caliper (Skin Caliper 3000, Dr. No, Germany), applied according to standard skinfold measurement procedures.

#### 2.3.2 Amputees' Mobility and RL Characteristics

The AAS [27] was used to assess the degree of mobility. A total value ranging between −50 and +70 points (pts.) can be achieved, corresponding to a certain degree of activity—high positive scores correlate positively with a high degree of mobility [27]. Furthermore, the overall mobility of each participating amputee was classified into Medicare's Functional Classification Level (MCFL, K‐Levels) [28], a five‐level functional classification system (K‐level 0–4) that quantifies the need and potential benefit of prostheses for patients after major amputation of the lower extremity.

Demographic data, level and cause of amputation were extracted from the Measurement and Classification of Amputation Stumps, ISPO (International Society for Prosthetics and Orthotics) 1982 [29] used in clinical routine.

#### 2.3.3 Gait Test

6MWT [30] was performed on even and stable ground. The subjects were asked to walk up and down a 20‐m‐long hallway for 6 min at their chosen maximum safe walking speed. Subjects were allowed to rest during the test and to resume walking as soon as they felt able to do so. At the end of the test, the total distance walked (in meters) was recorded.

### 2.4 Statistics

Demographics were assessed and evaluated in tabular form as master data (Microsoft Excel, Version 365 Enterprise, Microsoft, USA). For each questionnaire, the mean values of all total scores were calculated. Based on the master data, further scales could be listed that relate to specific characteristics, such as anthropometric data.

Both for LCF and SCFT, paired *t*‐tests were carried out to compare sound and residual limb at each measured location. Because three anatomical locations were tested (F1, F2, AL), significance was defined as *p* < 0.016 (Bonferroni correction: 0.05/3).

For the hyperspectral parameters (StO_2_, THI, TWI, NIRPI), statistical analyses were performed separately for each parameter and each location. A two‐way repeated‐measures ANOVA was applied with the within‐subject factors time point (Pre vs. Post Exercise) and leg (Sound vs. Residual Limb). As above, main effects and interactions were considered significant at *p* < 0.016, reflecting correction for the three locations.

If the interaction between leg and time point was significant, post hoc paired *t*‐tests were performed to compare Pre versus Post Exercise within each leg. Because two post hoc comparisons were performed for each of the three locations, the Bonferroni‐corrected threshold was *p* < 0.008 (0.05/6).

## 3 Results

### 3.1 Demographics

Ten male subjects (*n* = 10) with unilateral major lower limb amputation after trauma, tumor or infections were included in this study. Four subjects were TT and six were TF amputees. The mean age was 54.6 years (range: 29–77 years). The mean K‐Level was 3.1 ± 0.9, and the mean Amputee Activity Score (AAS) was +11 ± 19 points. Further, a mean walking distance of 324 ± 121 m was recorded (Table 1).

**TABLE 1:** Demographics of the included ten male probands, indicating the age, time since amputation, etiology and level of amputation.

	#0	#1	#2	#3	#5	#6	#7	#8	#9	#10	Mean
Age (years)	37	37	62	37	72	29	77	60	72	65	54.8 ± 17.9
Etiology	TU	T	T	T	I	T	TU	T	I	T	—
Years since amputation	3	13	3	7	2	3	1	1	6	12	5.1 ± 4.4
Level of amputation	TT	TT	TT	TT	TF	TF	TF	TF	TF	TF	—
Smoker	No	Yes	No	Yes	No	Yes	No	No	No	No	—
6MWT (m)	420	560	330	350	270	390	180	210	170	360	324 ± 121
K‐Level (0–4)	4	4	4	4	2	4	3	2	2	2	3.1 ± 1
AAS (pts.)	33	34	29	29	−15	12	−9	5	3	−11	11 ± 19

*Note:* Furthermore, it shows smoking status and the results of the 6‐min walk test as indicator for the endurance. Results display in meters, the K‐Level (1–4) and result of the Amputee Activity Score (AAS).

Abbreviations: I = infection, T = traumatic amputation, TU = tumor related amputation.

### 3.2 Anthropometric Data

**FIGURE 2:**
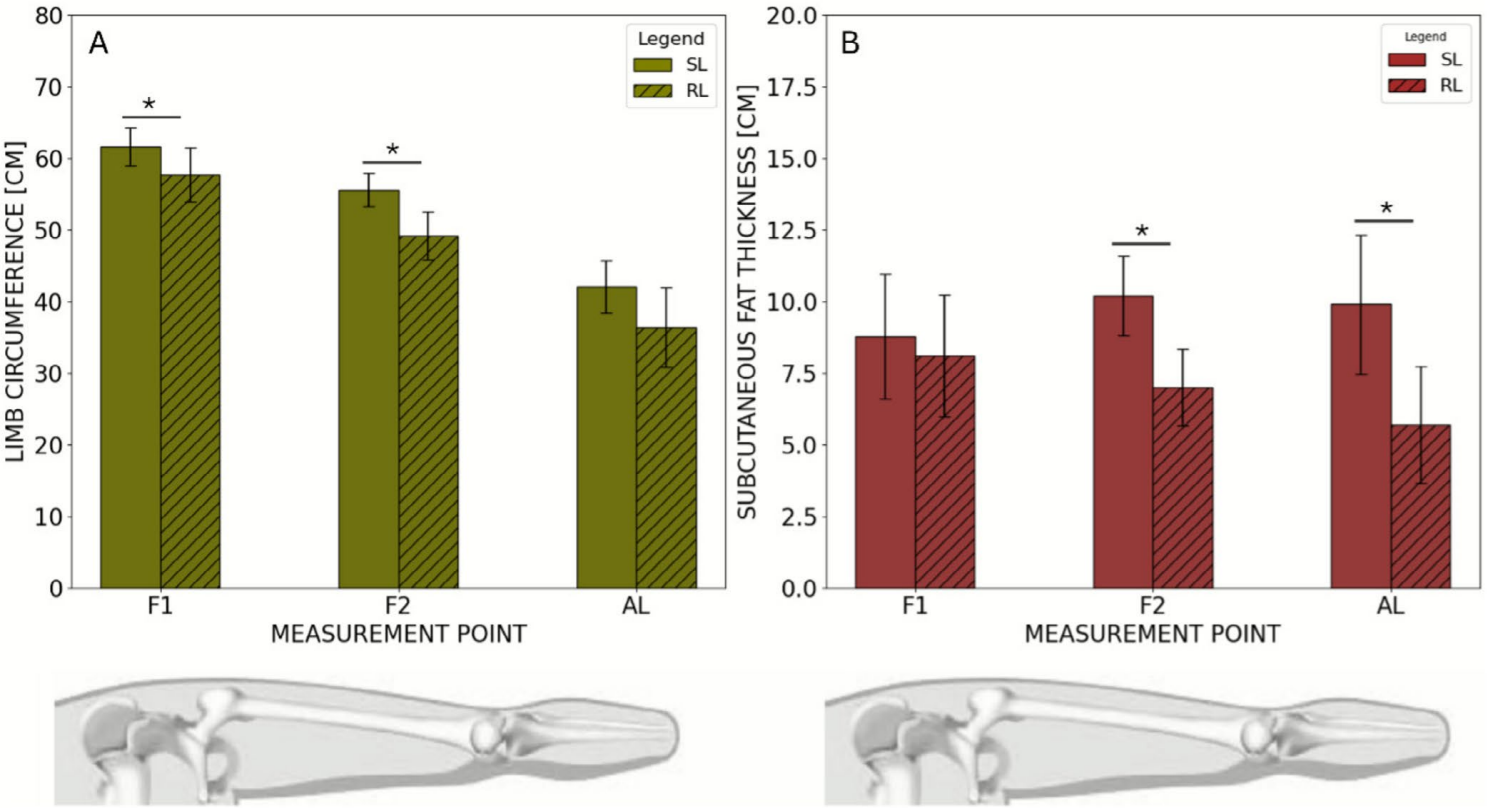
Comparison of the mean values between SL and RL of LCF (A) and SCFT (B). A significant reduction in LCF (absolute greatest difference at F2: 6.37 ± 3.63 cm) and SCFT (absolute greatest difference at AL: 4.2 ± 4.3 cm) was detected in the RL compared to the SL. * indicates *p* < 0.05.

Limb circumference (LCF) was significantly smaller at the residual limb at F1 (SL: 61.6 ± 2.7 cm; RL: 57.7 ± 3.8 cm; *p* = 0.0001) and F2 (SL: 55.6 ± 2.3 cm; RL: 49.2 ± 3.4 cm; *p* = 0.0004), with a similar trend at AL (SL: 42.0 ± 3.6 cm; RL: 36.3 ± 5.6 cm; *p* = 0.019; Figure 2A). Similarly, subcutaneous fat thickness (SCFT) was reduced at the residual limb, reaching significance at F2 (SL: 10.2 ± 1.4 mm; RL: 7.0 ± 1.4 mm; *p* = 0.015) and AL (SL: 9.9 ± 2.4 mm; RL: 5.7 ± 2.0 mm; *p* = 0.014; Figure 2B).

### 3.3 Hyperspectral Data

**FIGURE 3:**
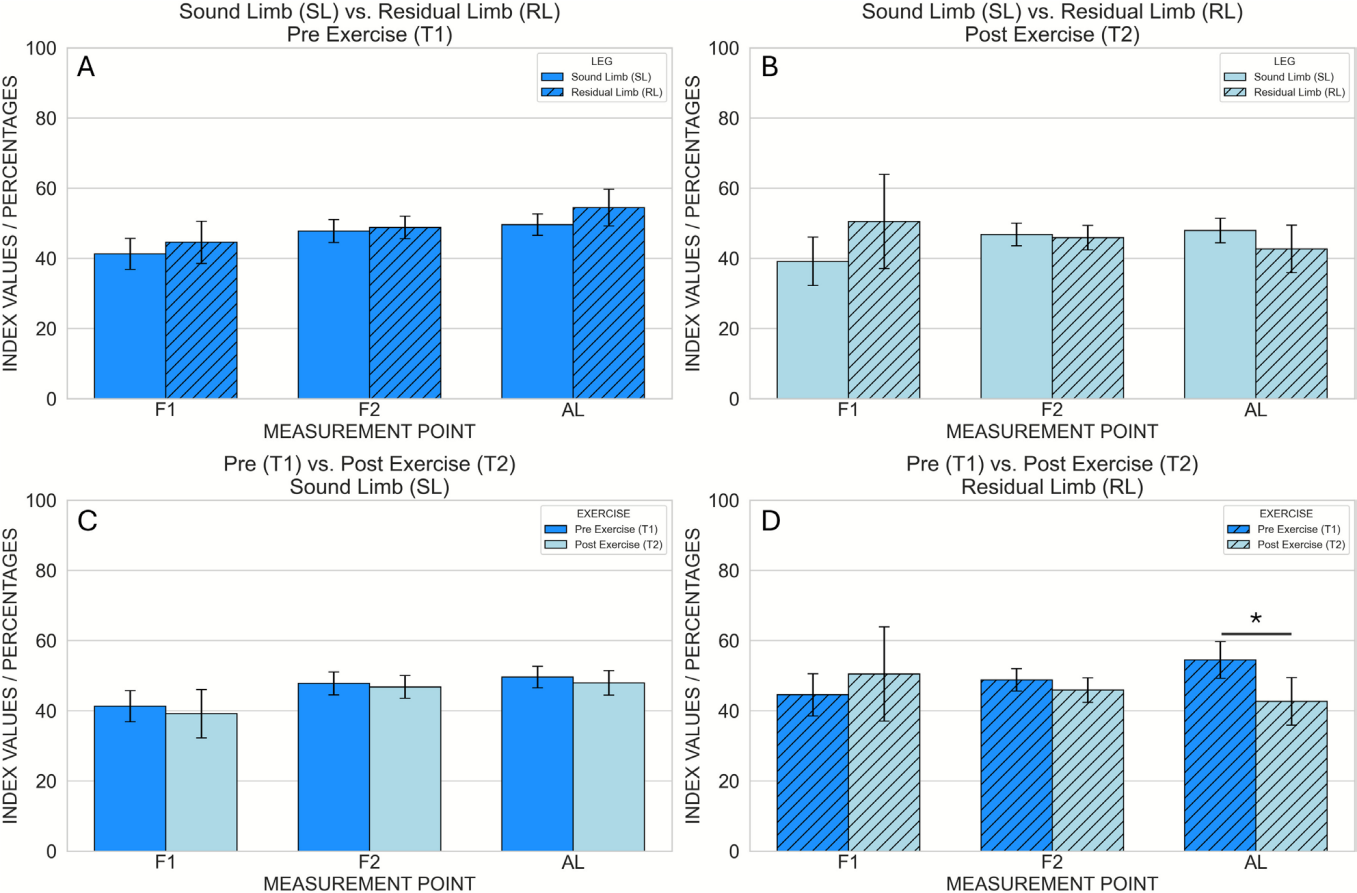
Hyperspectral StO_2_ (oxygen saturation) values measured at the three anatomical locations (F1, F2, AL). Comparison between Sound Limb and Residual Limb at Pre Exercise (A) and Post Exercise (B). Comparison between Pre Exercise and Post Exercise at the Sound Limb (C) and Residual Limb (D). Error bars represent the intrasubject standard deviation. An asterisk (*) indicates significant pairwise differences after Bonferroni correction (*p* < 0.008).

**FIGURE 4:**
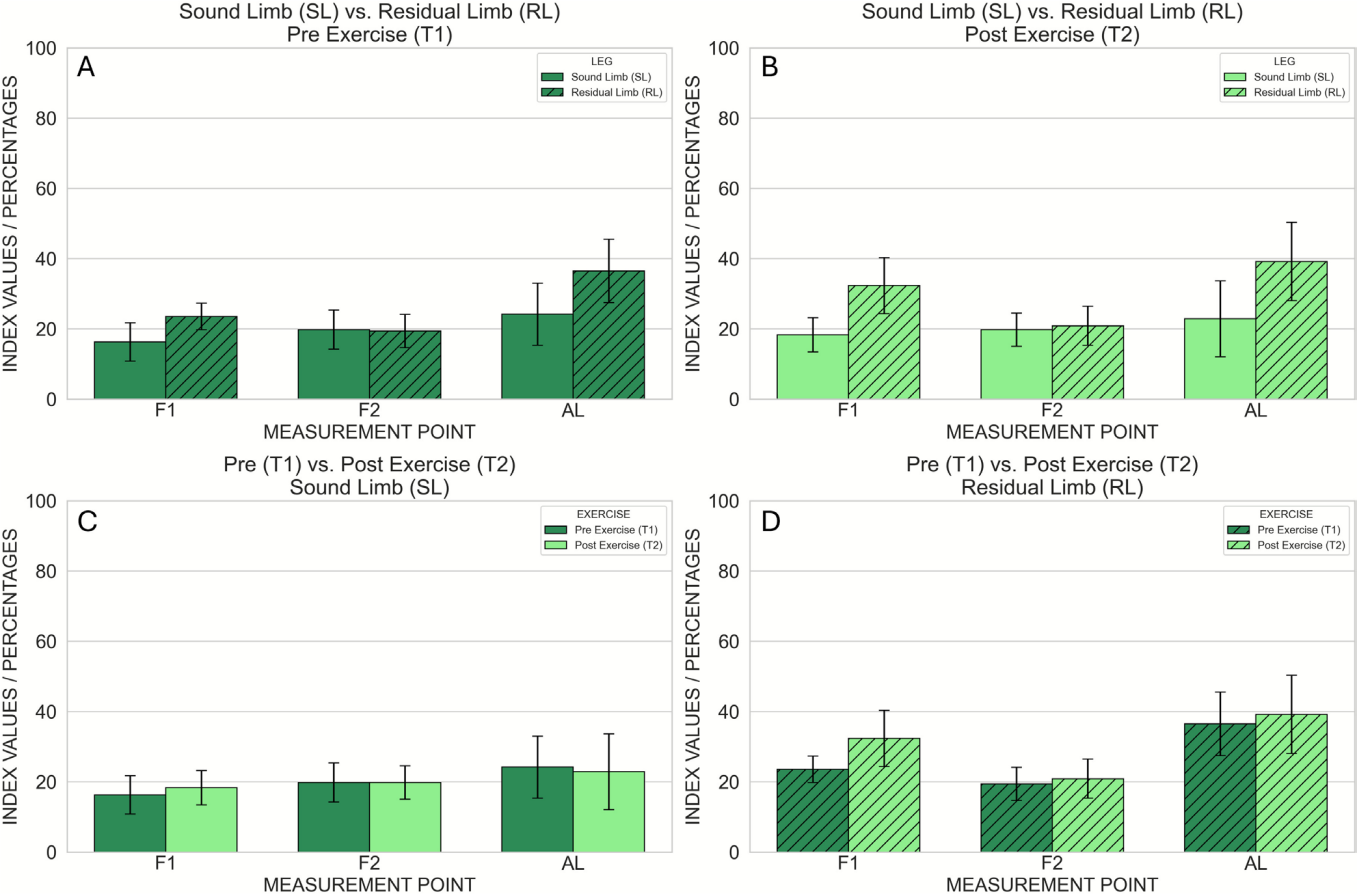
Hyperspectral THI (tissue hemoglobin index) values measured at the three anatomical locations (F1, F2, AL). Comparison between Sound Limb and Residual Limb at Pre Exercise (A) and Post Exercise (B). Comparison between Pre Exercise and Post Exercise at the Sound Limb (C) and Residual Limb (D). Error bars represent the intrasubject standard deviation.

**FIGURE 5:**
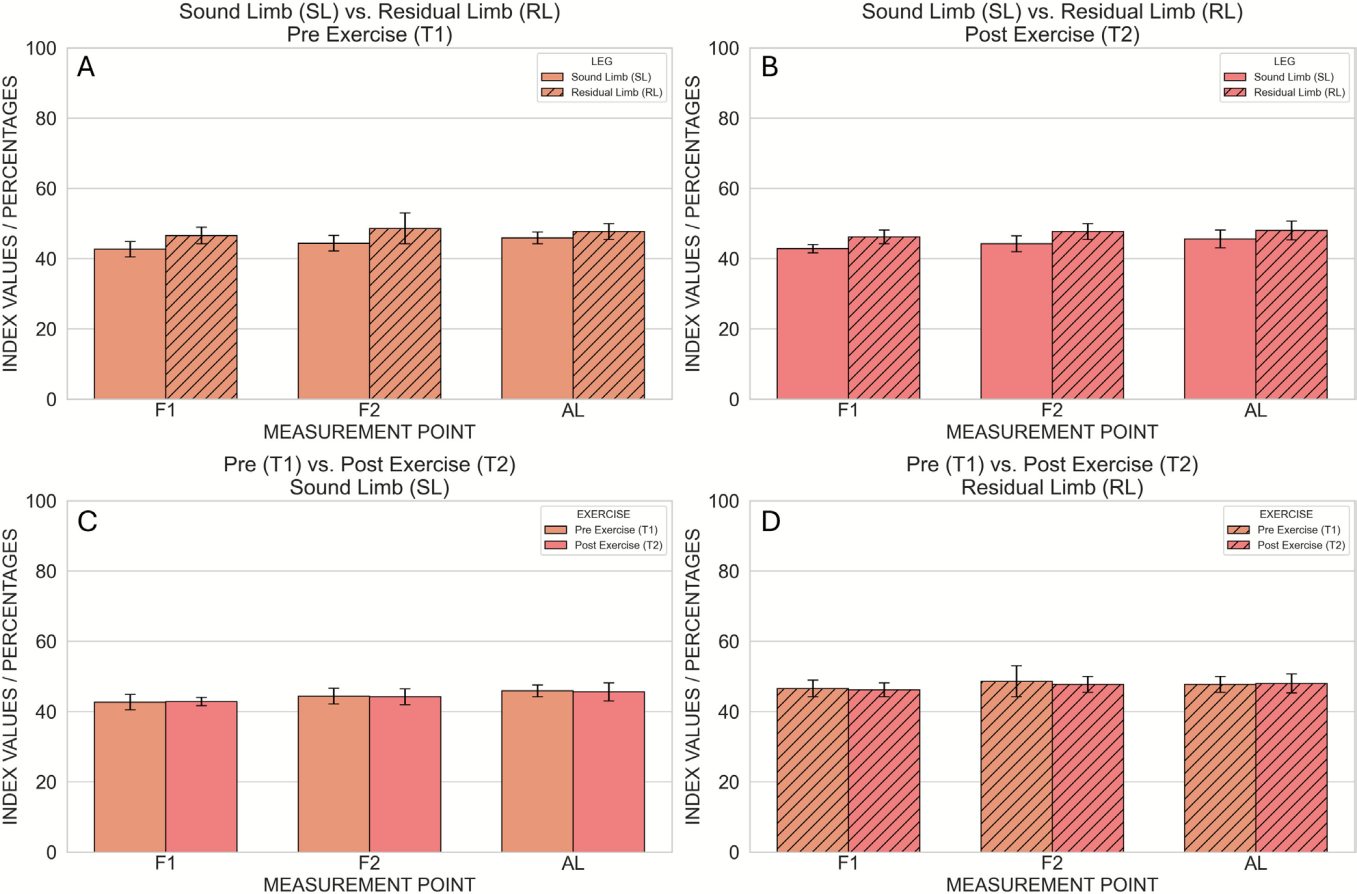
Hyperspectral TWI (tissue water index) values measured at the three anatomical locations (F1, F2, AL). Comparison between Sound Limb and Residual Limb at Pre Exercise (A) and Post Exercise (B). Comparison between Pre Exercise and Post Exercise at the Sound Limb (C) and Residual Limb (D). Error bars represent the intrasubject standard deviation.

**FIGURE 6:**
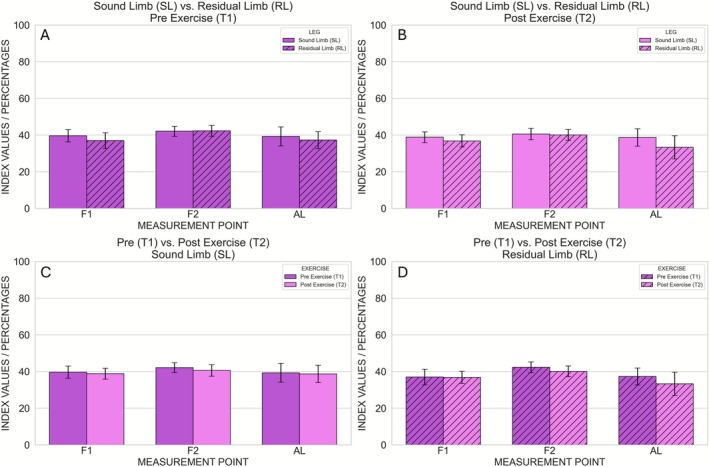
Hyperspectral NIRPI (near‐infrared perfusion index) values measured at the three anatomical locations (F1, F2, AL). Comparison between Sound Limb and Residual Limb at Pre Exercise (A) and Post Exercise (B). Comparison between Pre Exercise and Post Exercise at the Sound Limb (C) and Residual Limb (D). Error bars represent the intrasubject standard deviation.

**FIGURE 7:**
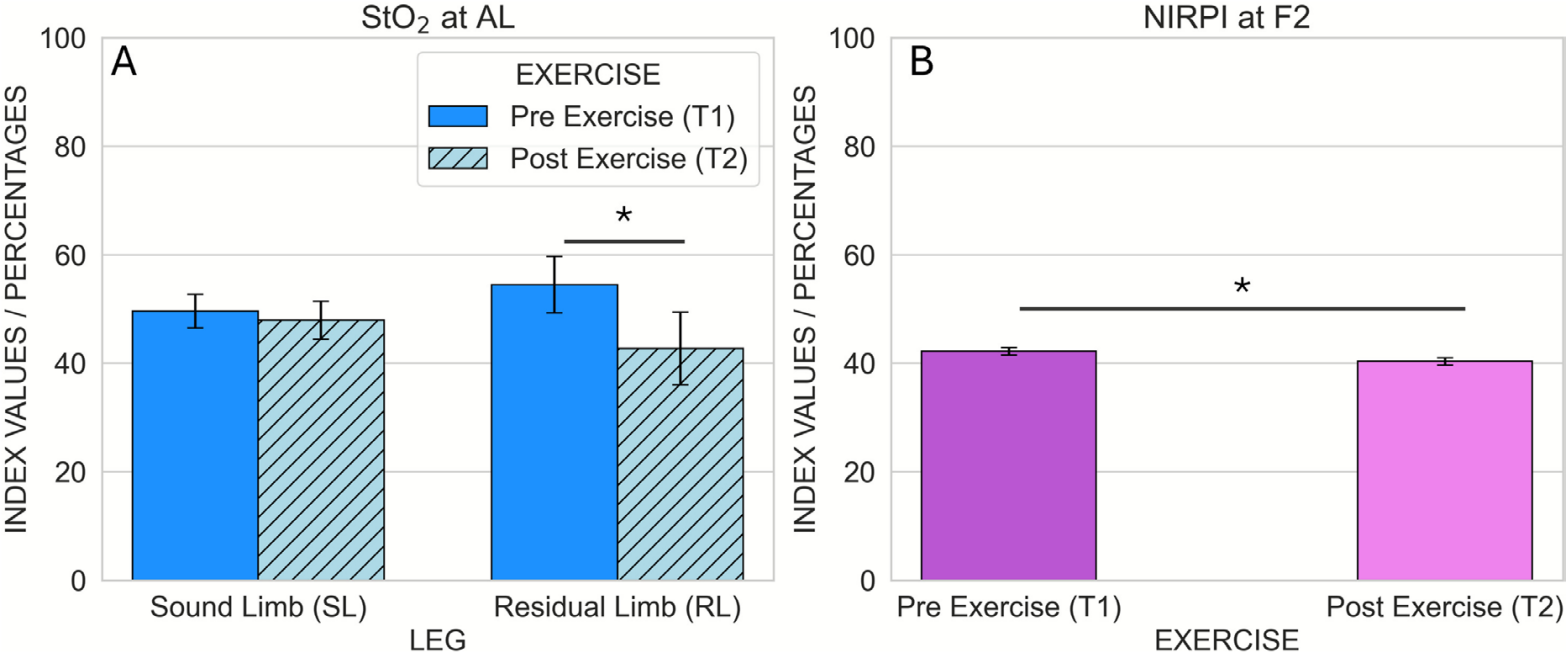
Significant results for StO_2_ at AL and NIRPI at F2. Results for the StO2 condition, separated by Leg (Residual and Sound Limb) and Time Point (Pre and Post Exercise) (A) and for the NIRPI condition, separated by Time Point (Pre and Post Exercise) (B). Error bars represent the intrasubject standard deviation. An asterisk (*) indicates significant pairwise differences after Bonferroni correction (*p* < 0.008).

The hyperspectral mean values and intrasubject standard deviations for each parameter and measurement location are shown in Figures 3, 4, 5, 6, 7, and the corresponding inferential statistics are summarized in Table S1. A representative example of the hyperspectral maps is provided in Figure S1.

After correcting for the three measurement locations per parameter, two statistically significant effects were observed. First, NIRPI at F2 was significantly higher before exercise than after exercise (Pre exercise: 42.2 ± 0.7; Post exercise: 40.4 ± 0.7), as indicated by a main effect of time point (*p* = 0.0016) without a significant leg × time interaction (Figures 6 and 7B). Second, at AL the main effect of time point on StO_2_ was significant (*p* = 0.0096), and this was accompanied by a significant leg × time interaction (*p* = 0.010). Post hoc paired *t*‐tests showed that, in the sound limb, StO_2_ did not differ significantly between Pre and Post Exercise (49.6 ± 3.1 vs. 47.9 ± 3.5; *p* = 0.2002), whereas in the residual limb StO_2_ was significantly higher before exercise (54.5 ± 5.2) than after exercise (42.7 ± 6.7; *p* = 0.0074) (Figures 3 and 7A). No other comparisons survived correction for multiple testing, and no significant pre–post differences in StO_2_, NIRPI, THI or TWI were observed in the sound limb.

## 4 Discussion

The aim of this study was to investigate and analyze the microcirculation of the RL of non‐dysvascular major lower limb amputation in comparison to the SL at rest (T1) and after walking in a socket prosthesis (T2).

As expected, the limb circumferences and subcutaneous fat thickness were reduced for almost all measured locations from the SL to the RL.

HSI uses color‐coding and allows for a planar, simultaneous imaging of tissue surfaces to detect possible pathologies [19, 31, 32, 33]. A region of interest can be chosen for detailed results given in percentage or index values of any location within the color‐coded field.

Our results demonstrated that the NIRPI was significantly decreased by roughly 5% after walking in a socket prosthesis at F2. However, as there was no significant interaction between leg and time point for NIRPI, this reduction must have been similar for both limbs. In contrast, the significant STO_2_ interaction at AL between leg and time point turned out to be due to a significantly 22%‐ reduction in StO_2_ at T2 compared to T1 only at the residual limb. These findings indicate that walking in a socket prosthesis significantly impacts the oxygen saturation in the residual limb but not in the sound limb.

Other studies have also demonstrated that muscular activity can cause a reduced oxygen level [34, 35] as a result of oxygen consumption. Herein, the oxygen saturation of superficial tissue at the distal RL was significantly lower when compared to the SL after walking in a socket prosthesis. Although previous research could show overall higher energy consumption during ambulation of amputees [36], oxygen distribution has, to our knowledge, never been analyzed in an intra‐individual comparison of the SL and RL. Furthermore, this finding underlines the assumption of socket‐induced stress at the distal zones of the RL even though sockets should prevent weight bearing to avoid skin breakdown and pain.

The crucial question remains if the containment of the socket exceeds critical values of 25 mmHG on the skin capillaries at certain locations at the RL during walking. Another study investigated TcPO_2_ levels at the RL at rest with and without wearing a liner [37]. That results revealed impaired microcirculation over the tip of the tibia end even at resting position by local pressure induced by wearing a liner solely [38].

In this study, TWI and THI showed numerically higher values at the RL than at the SL at several measurement locations (F1 and F2 for TWI; F1 and AL for THI). In hyperspectral imaging, elevated TWI values reflect increased superficial water content, which can be consistent with edema; however, these differences did not reach statistical significance after correction for multiple testing. As a result, no physiological conclusions, such as edema, venous stasis, or impaired lymphatic drainage, can be drawn from the present dataset.

Nevertheless, it is important to note that alterations in TWI and THI have been associated with clinically relevant residual‐limb phenomena in earlier work. Volume instability of the RL, which may arise from disrupted venous and lymphatic systems after amputation, has been frequently reported in the literature [39, 40, 41]. In other clinical contexts, hyperspectral imaging has demonstrated that decreased StO_2_ and TWI together with increased THI can indicate compromised microcirculation or venous outflow obstruction and may even serve as early markers for tissue compromise in free‐flap monitoring [31]. THI in particular has been proposed as a parameter capable of distinguishing venous congestion from arterial inflow problems [13, 31, 42, 43], while decreased THI and StO_2_ have been described as characteristic for arterial inflow disorders [21, 43].

Overall, the classification to K‐Levels and performance results indicate that the cohort within this study belongs to a highly active subset of the amputee population. The recruitment of only men limits the generalizability of this physiological study. The recruitment result is due to the fact that the prevalence of males is reported to be three times higher compared to female non‐dysvascular amputees [44]. Therefore, future studies should include gender equality.

Additional limitations of this study are that the socket design was not standardized and that pressure in the socket at defined locations was not measured when walking with the socket prosthesis. Still, the significant difference of the oxygen parameters (StO_2_) at the RL at T2 in comparison to unaffected oxygen parameters of the SL supports the assumptions that socket‐related parameters at the RL in addition to oxygen consumption during physical activity might be a relevant factor for decreased oxygen levels as there were no changes at the SL after physical activity.

Unfortunately, for TTs only one measurement point (AL) was exposed to pressure by the socket at T2. For TF, in contrast, all measurement points were located inside the prosthetic socket (AL, F1, F2) and exposed to pressure by the socket at T2. Despite this, we pooled for statistical analysis socket‐exposed measurements of F1 and F2 in TFs with F1 and F2 data of TTs that were not exposed to the pressure of the socket.

Nevertheless, the planar color‐coded HSI images (Supporting Information) visualized regional differences at the SL and RL after walking (T2). The results are in accordance with our clinical observations of amputees' requirements and the reality of current socket design. To find the balance between secure suspension of the socket as a prerequisite for safe walking on the prosthetic components and pain‐free comfort remains up to now challenging. Anyway, the message of these findings should not be that physical activity after amputation should be avoided.

We should be encouraged to focus on residual limb health determined by its perfusion, tissue mechanics and overall limb morphology. Limb volume and perfusion mainly drive socket fit as a prerequisite for secure walking and skin integrity [45, 46]. The finding of this study might advocate a dynamic approach for socket design, avoiding capillary occlusion, critical oxygenation, edema, and venous stasis to prevent skin breakdown and support residual limb health [13, 43, 47].

Current 3D scanning and traditional casting are done on a resting RL. Although sockets target to enable movement, the standard static approach does not respect the movement of the different tissues at the RL when walking with the socket prosthesis [45].

The size and heterogeneity of amputation levels within this study design do not allow correlating differences of microcirculatory and anthropometric parameters with performance and pain. If the gait speed of different amputation levels is related to the biomechanical implications of the amputation level rather than oxygen supply, it should be investigated in future studies [48, 49, 50, 51].

## 5 Conclusion

In this study we evaluated anthropometric data (LCF, SCFT), mobility and microcirculatory parameters (StO_2_, NIRPI, THI, TWI) measured with hyperspectral imaging (TIVITA Tissue Diaspective Vision, Germany) of ten lower leg non‐dysvascular transtibial (*n* = 4) and transfemoral (*n* = 6) amputees. Hyperspectral data were measured at rest (T1) and after physical activity (6MWT) (T2) at defined localizations (F1, F2, AL) of both legs (SL, RL).

Regarding microcirculation, only two findings reached statistical significance: a general decrease in NIRPI after walking at F2, and a localized post‐exercise reduction in StO_2_ at the residual limb at AL. No other microcirculatory parameters, including TWI and THI, differed significantly between limbs or across time after correction for multiple comparisons.

These results demonstrate that hyperspectral imaging can detect localized oxygenation changes in the residual limb following activity, while other parameters did not show significant alterations in this small cohort. The observed numerical differences in THI and TWI may warrant investigation in larger samples to determine whether underlying microcirculatory patterns can be confirmed. Finally, given that only the residual limb showed an exercise‐related decrease in StO_2_, future studies should examine how socket fit, tissue loading, and dynamic limb behavior contribute to residual limb perfusion during gait.

## 6 Perspective

Our findings support the importance of microcirculation for residual limb health. The perspective could be a smart, auto‐adaptive socket that abandons the principles of the current static and rigid approach to socket design and fabrication. This new design would avoid capillary occlusion, ensure critical oxygenation, and respect issues such as edema and venous stasis to prevent skin breakdown and support overall residual limb health. The results indicate that hyperspectral imaging (HSI), as a non‐radiative and near real‐time modality for microcirculation imaging, might allow for the semiquantitative analysis of microcirculatory parameters of the RL.

## Funding

The MOBILISE‐N research group, within which this study was conducted, receives funding from Ottobock SE & Co. KGaA.

## Ethics Statement

This study was conducted ethically in accordance with the World Medical Association Declaration of Helsinki and reviewed as well as approved by the local ethics committee, approval number 26/03/18.

## Consent

The patients gave their written informed consent.

## Conflicts of Interest

The authors declare no conflicts of interest.

## Supporting information


**Table S1:** Statistical results of the two‐way repeated measures ANOVAs conducted for each measure (STO_2_, THI, TWI, and NIRPI) at each of the three leg locations. The factors considered were time point (T1 vs. T2) and leg (SL vs. RL). Significance was determined at *p* < 0.016, that is, correcting for the three locations. For significant interactions between leg and time point, post hoc paired *t*‐tests were performed to compare time points separately for SL and RL, with significance set at *p* < 0.008, correcting for the two tests and three locations. The results include the *F*‐values and *p*‐values for each main effect and interaction.
**Figure S1:** Color‐coded HSI false‐color images of the RL at time points T1 and T2. Exemplary imaging of one transfemoral amputees: The parameters StO_2_ (A), NIRPI (B), THI (C) and TWI (D) of the RL are compared at T1 (left) and T2 (right). The red circles mark the three measurement locations (F1, F2, AL) from proximal to distal. The numbers correspond to the measured percentage as index values. The color‐coding is used for visualization: blue shades correspond to low percentage or index values, green shades to medium percentage or index values and red shades to high percentage or index values. Further color‐coded HSI false‐color images of each included proband (*n* = 9) can be found in supplementary data.

## Data Availability

Datasets are available on request.
