# Supplementary material for: Hyperspectral Imaging Reveals High Water and Hemoglobin Content at Rest and Decreased Oxygen Levels After Physical Activity at the Residual Limb of Non‐Dysvascular Lower Limb Amputees
Source: Microcirculation. 2026 Jan 18;33(1):e70051. doi: 10.1111/micc.70051 (PMC12812306; doi:10.1111/micc.70051)
Supplement: Supplementary file 1 — Table S1: Statistical results of the two‐way repeated measures ANOVAs conducted for each measure (STO2, THI, TWI, and NIRPI) at each of the three leg locations. The factors considered were time point (T1 vs. T2) and leg (SL vs. RL). Significance was determined at p < 0.016, that is, correcting for the three locations. For significant interactions between leg and time point, post hoc paired t‐tests were performed to compare time points separately for SL and RL, with significance set at p < 0.008, correcting for the two tests and three locations. The results include the F‐values and p‐values for each main effect and interaction. Figure S1: Color‐coded HSI false‐color images of the RL at time points T1 and T2. Exemplary imaging of one transfemoral amputees: The parameters StO2 (A), NIRPI (B), THI (C) and TWI (D) of the RL are compared at T1 (left) and T2 (right). The red circles mark the three measurement locations (F1, F2, AL) from proximal to distal. The numbers correspond to the measured percentage as index values. The color‐coding is used for visualization: blue shades correspond to low percentage or index values, green shades to medium percentage or index values and red shades to high percentage or index values. Further color‐coded HSI false‐color images of each included proband (n = 9) can be found in supplementary data. [file MICC-33-e70051-s001.docx]

# **Supplementary material**

| Measure | Location | Main effect/ Interaction | F-value | p-value |
| --- | --- | --- | --- | --- |
| STO2 | F1 | Leg | 3.32 | 0.1279 |
|  |  | Time point | 1.17 | 0.3279 |
|  |  | Leg x time point | 0.68 | 0.4483 |
|  | F2 | Leg | 0 | 0.9778 |
|  |  | Time point | 3.73 | 0.0855 |
|  |  | Leg x time point | 3.42 | 0.0973 |
|  | AL | Leg | 0.01 | 0.9305 |
|  |  | Time point | 10.75 | **0.0096*** |
|  |  | Leg x time point | 10.57 | **0.0100*** |
| THI | F1 | Leg | 10.14 | **0.0244** |
|  |  | Time point | 2.89 | 0.1499 |
|  |  | Leg x time point | 1.71 | 0.2478 |
|  | F2 | Leg | 0.01 | 0.912 |
|  |  | Time point | 0.77 | 0.4026 |
|  |  | Leg x time point | 1.22 | 0.2987 |
|  | AL | Leg | 5.5 | **0.0436** |
|  |  | Time point | 0.75 | 0.4101 |
|  |  | Leg x time point | 1.83 | 0.2094 |
| TWI | F1 | Leg | 9.23 | **0.0288** |
|  |  | Time point | 1 | 0.3632 |
|  |  | Leg x time point | 1.43 | 0.2856 |
|  | F2 | Leg | 7.8 | **0.021** |
|  |  | Time point | 0.37 | 0.5597 |
|  |  | Leg x time point | 0.17 | 0.6901 |
|  | AL | Leg | 3.08 | 0.1132 |
|  |  | Time point | 0 | 1 |
|  |  | Leg x time point | 0.35 | 0.5683 |
| NIRPI | F1 | Leg | 0.85 | 0.3991 |
|  |  | Time point | 0.43 | 0.5416 |
|  |  | Leg x time point | 0.1 | 0.7679 |
|  | F2 | Leg | 0.01 | 0.935 |
|  |  | Time point | 19.84 | **0.0016*** |
|  |  | Leg x time point | 2 | 0.1914 |
|  | AL | Leg | 1.43 | 0.2617 |
|  |  | Time point | 5.87 | **0.0384** |
|  |  | Leg x time point | 4.9 | 0.0542 |

**Supplementary Table 1: Statistical results of the two-way repeated measures ANOVAs conducted for each measure (STO2, THI, TWI, and NIRPI) at each of the three leg locations.** The factors considered were time point (T1 vs. T2) and leg (SL vs. RL). Significance was determined at p < 0.016, i.e. correcting for the three locations. For significant interactions between leg and time point, post-hoc paired t-tests were performed to compare time points separately for SL and RL, with significance set at p < 0.008, correcting for the two tests and three locations. The results include the F-values and p-values for each main effect and interaction.

**Supplementary Figure 1:** **Color-coded HSI false-color images of the RL at time points T1 and T2.** Exemplary imaging of one transfemoral amputees: The parameters StO2 (A), NIRPI (B), THI (C) and TWI (D) of the RL are compared at T1 (left) and T2 (right). The red circles mark the three measurement locations (F1, F2, AL) from proximal to distal. The numbers correspond to the measured percentage as index values. The color-coding is used for visualization: blue shades correspond to low percentage or index values, green shades to medium percentage or index values and red shades to high percentage or index values. Further color-coded HSI false-color images of each included proband (n = 9) can be found in supplementary data.
